# Ketogenic Diet, Serum Ketone Bodies and Risk of End‐Stage Renal Disease in Patients With Diabetic Kidney Disease: A Multi‐Cohort Study

**DOI:** 10.1111/1753-0407.70140

**Published:** 2025-08-12

**Authors:** Ke Liu, Qing Yang, Yanlin Lang, Yutong Zou, Jiamin Yuan, Jia Yang, Jing Ma, Linli Cai, Xianglin Kong, Fuhai Yang, Fang Liu

**Affiliations:** ^1^ Department of Nephrology West China Hospital of Sichuan University Chengdu China; ^2^ Laboratory of Diabetic Kidney Disease, Kidney Research Institute, Department of Nephrology West China Hospital, Sichuan University Chengdu China; ^3^ Longmatan District People's Hospital Luzhou China; ^4^ Shidong Street Community Health Service Center Luzhou China

**Keywords:** diabetic kidney disease, end‐stage renal disease, ketogenic diet, ketone body, β‐Hydroxybutyrate

## Abstract

**Aim:**

This study aims to explore the effect of the ketogenic diet (KD) on the occurrence of end‐stage renal disease (ESRD) and the longitudinal relationship between circulating β‐hydroxybutyrate (β‐OHB) and kidney outcomes.

**Methods:**

We used the dietary ketogenic ratio (DKR) to estimate the nutritional ketosis probability of KD and analyzed the association with ESRD using NHANES cross‐sectional data by Spearman correlation coefficient and multivariate logistic regression model. We also used the Kaplan–Meier method, Cox regression analysis, and restricted cubic splines (RCS) to analyze the relationship between circulating β‐OHB and renal outcomes in the T2DM‐DKD longitudinal cohort of West China Hospital. Mendelian randomization (MR) was also employed to evaluate potential causal associations.

**Results:**

The cross‐sectional analysis revealed that non‐ESRD patients had significantly higher baseline age, BMI, serum albumin, and DKR values, with a weak negative correlation between DKR and serum creatinine (*ρ* = −0.072, *p* = 0.011). Logistic regression consistently indicated a reduced ESRD prevalence in higher DKR quartiles. In the longitudinal study, elevated β‐OHB levels were associated with improved renal survival and a lower risk of ESRD, with RCS analysis identifying the lowest risk at approximately 0.25 mmol/L. MR analyses supported these findings, showing inverse correlations between genetically predicted β‐OHB and creatinine (*p* = 0.007) and cystatin c (*p* < 0.001).

**Conclusion:**

These findings suggest that KD may be associated with a lower incidence of ESRD in DKD patients, with elevated β‐OHB levels independently associated with a reduced risk of ESRD, warranting further research to confirm causality and elucidate underlying mechanisms.

## Graphical abstract

Ketogenic diet (KD) promotes nutritional ketosis and raises β‐hydroxybutyrate (β‐OHB) levels. Using cross‐sectional, longitudinal, and Mendelian randomization analyses, we found that elevated β‐OHB levels were independently associated with a reduced risk of ESRD. A nonlinear relationship between β‐OHB and ESRD risk was observed in DKD patients, suggesting that maintaining high‐normal β‐OHB levels may help delay DKD progression.

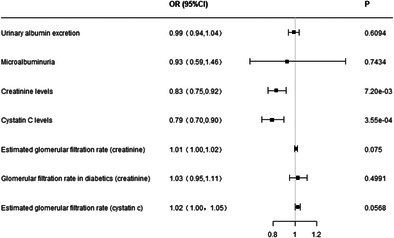

**Summary**

- Our study found that a ketogenic diet (via dietary ketogenic ratio) correlates with serum creatinine and ESRD incidence in DKD.
- A nonlinear association between β‐OHB and ESRD risk was observed in ketosis‐free DKD patients, independent of confounders.
- Maintaining high‐normal β‐OHB levels may mitigate DKD progression in T2DM, and MR analyses supported these findings.

## 1 Background

Over the past 30 years, the prevalence of diabetes in China has surged from under 1% to roughly 12% due to a combination of factors such as population growth and aging, changes in dietary structure, reduced physical activity, increased awareness and management of diabetes, and changes in diagnostic criteria [1, 2]. Diabetic kidney disease (DKD) is a common microvascular complication of type 2 diabetes mellitus (T2DM), resulting from hyperglycemia‐induced disturbances in tubulo‐glomerular feedback and direct glucotoxicity, with a reported prevalence of 21.8% in Chinese T2DM patients [3, 4]. Early identification of biomarkers and risk factors is thus critical to preventing DKD progression.

Ketone bodies (KB)—β‐hydroxybutyrate (β‐OHB, or 3‐hydroxybutyrate), acetoacetate, and acetone—are produced in the liver via fatty acid oxidation and serve as alternative energy sources during fasting or exercise [5]. Beyond energy provision, acts as a signaling molecule that influences pathways involved in obesity, diabetes, aging, cardiovascular disease, and cancer [6, 7]. Its production is regulated by glucagon and insulin, and dysregulated glycemic control can alter KB metabolism. In diabetes, elevated KB are linked to complications such as renal injury, activation of the renin‐angiotensin‐aldosterone system, reduced autophagy, and increased renal fibrosis via the transforming growth factor‐β (TGF‐β)/mothers against decapentaplegic homolog 3 (Smad3) pathway, though their role in chronic complications remains unclear [8, 9].

Recent evidence suggests that moderate elevations of β‐OHB may be renoprotective. Experimental studies indicate that β‐OHB can inhibit mechanistic target of rapamycin complex 1 (mTORC1) in proximal tubular cells and podocytes, and sodium‐glucose co‐transporter 2 inhibitors (SGLT2i)—which increase endogenous KB—demonstrate renal protection [10, 11]. Moreover, cross‐sectional data reveal a J‐shaped relationship between circulating KB and DKD risk in ketosis‐free T2DM patients, with high‐normal β‐OHB levels correlating weakly with eGFR and possibly slowing DKD progression [12]. However, longitudinal and genetic studies are needed to clarify these associations.

The ketogenic diet (KD), defined by low carbohydrate, high fat, and moderate protein intake, shifts metabolism from carbohydrates to fats, inducing nutritional ketosis with elevated β‐OHB and acetoacetate levels [13]. KD has been associated with improved insulin sensitivity, reduced inflammation, and enhanced cognitive function, and preliminary studies suggest that a very low‐calorie KD may improve renal filtration markers and mitigate risk factors for DKD [14, 15, 16].

In this study, we analyze the association between KD and end‐stage renal disease (ESRD) incidence in DKD patients. By retrospectively examining the relationship between the circulation β‐OHB level and DKD progression, we aim to identify the optimal β‐OHB range that minimizes ESRD risk. Furthermore, two‐sample Mendelian randomization (MR) will be employed to investigate the causal link between circulating 3‐hydroxybutyrate and renal function markers.

## 2 Method

We applied complementary study designs: (1) a cross‐sectional analysis of the association between KD and ESRD incidence in the DKD population using dietary data from the National Health and Nutrition Examination Survey (NHANES) database, (2) a longitudinal observational study of serum β‐OHB on the progression of DKD to ESRD in Chinese T2DM‐DKD patients, and (3) a two‐sample MR study using a genome‐wide association study (GWAS) of publicly available β‐OHB and kidney function biomarkers.

### 2.1 Population Studies

#### 2.1.1 Cross‐Sectional Study

##### 2.1.1.1 Study Design and Participants

We conducted a cross‐sectional study using NHANES (2003–2020) data, which includes dietary recalls and renal function tests (https://www.cdc.gov/nchs/NHANES/index). Initiated by the National Center for Health Statistics (NCHS) in 1999, NHANES assesses the health and nutrition of noninstitutionalized U.S. civilians. From 95 872 participants, we excluded those under 18 years old (*n* = 38 416), non‐diabetics (*n* = 36 242), individuals not meeting DKD criteria (*n* = 6989), and those with missing examination (*n* = 12 678) or covariate data (*n* = 280), yielding a final sample of 1267 adults. All participants provided written informed consent, and the study protocol was approved by the NCHS Ethics Review Committee.

##### 2.1.1.2 The Dietary Ketogenic Ratio (DKR)

Dietary intake was assessed via two 24‐h recalls conducted by trained interviewers. Nutrient intakes were computed using the Food and Nutrition database. The DKR was calculated using Withrow's equation: DKR = (0.9 × grams of fat + 0.46 × grams of protein)/(0.1 × grams of fat + 0.58 × grams of protein + grams of net carbohydrates) [17, 18]. This index ranges from 0 to 9, with higher values indicating a greater potential to induce nutritional ketosis.

##### 2.1.1.3 Outcome and Covariates

The definition of renal outcome was ESRD, defined as dialysis treatment or eGFR < 15 mL/min/1.73 m^2^ [19]. Covariates comprised age, sex, education level (less than high school vs. high school and above), smoking status, alcohol consumption, body mass index (BMI), blood lipids, serum albumin, and glycated hemoglobin. Missing covariate data (BMI, 4.1%; glycated hemoglobin, 0.6%; hypertension, 0.4%) were imputed using multiple imputation methods, with all missing proportions below 10%.

##### 2.1.1.4 Statistical Analysis

Participants were stratified by ESRD status. Continuous variables are presented as mean ± standard deviation (SD) or median (interquartile range), and categorical variables as counts and percentages. Group differences were evaluated using Student's *t*‐test for normally distributed continuous data, the Wilcoxon rank‐sum test for nonnormal distributions, and the Chi‐Square test for categorical variables. Pearson or Spearman correlation analyses were conducted to assess the relationship between DKR, eGFR, and urine albumin‐to‐creatinine ratio (UACR). Binary logistic regression estimated the association between DKR quartiles (Q1 as the reference) and ESRD, yielding odds ratios (ORs) with 95% confidence intervals (CIs). Three models with increasing adjustments were constructed. All analyses were performed using R version 4.4.1.

#### 2.1.2 Longitudinal Observational Study

##### 2.1.2.1 Patient Selection and Study Design

Between 2010 and 2019, we enrolled 432 patients with type 2 DKD diagnosed by kidney biopsy at West China Hospital of Sichuan University to form a follow‐up cohort. Exclusions were follow‐up < 1 year (*n* = 48), pre‐biopsy eGFR < 15 mL/min/1.73 m^2^ (*n* = 12), metabolic acidosis (serum bicarbonate < 15 mmol/L; *n* = 5), or missing data (*n* = 21), yielding 346 eligible patients. Diabetes is diagnosed based on American Diabetes Association criteria [20]. Indications for kidney biopsy in these T2DM patients included sudden significant albuminuria, rapid decline in renal function (with eGFR calculated using the CKD‐EPI equation [21]), absence of diabetic retinopathy, or presence of active urinary sediment. The pathological diagnosis of DKD was confirmed by at least two pathologists following the 2010 Society for Renal Pathology Classification [22]. All patients provided written informed consent, and this study was approved by the institutional review board at the West China Hospital of Sichuan University.

##### 2.1.2.2 Clinical and Pathological Features

Baseline clinical data were obtained by interview and anthropometrics, including sex, age, body mass index, duration of diabetes, blood pressure, dyslipidemia and medication history. Blood and urine samples were analyzed with a Cobas Intera 400 Plus (Roche, Basel, Switzerland) to measure creatinine, serum β‐OHB, triglycerides, cholesterol, HbA1c, fasting glucose, and 24‐h urine protein. The kidney outcome was defined by the progression to ESRD. Renal pathology specimens, obtained by needle biopsy, were evaluated using light microscopy, electron microscopy, and immunofluorescence according to the Renal Pathology Society classification.

##### 2.1.2.3 Statistical Analysis

It were performed using SPSS 27.0 (IBM Corp., Armonk, NY) and R software (version 4.4.1; R Foundation for Statistical Computing, Vienna, Austria), the latter for logistic models with restricted cubic splines (RCS). Statistical description is the same as above. Group differences were assessed using one‐way ANOVA, nonparametric tests, and Chi‐Square tests. Kidney survival was evaluated with Kaplan–Meier and log‐rank tests, while univariate and multivariate Cox analyses examined the relationship between β‐OHB and renal outcomes. RCS was used to explore the nonlinear association between β‐OHB levels and ESRD incidence. A two‐tailed *p* < 0.05 was considered statistically significant.

### 2.2 MR Study

Genetic variants for 3‐Hydroxybutyrate (GWAS ID: met‐d‐bOHbutyrate) were sourced from UK Biobank metabolic biomarker data. Summary‐level data for renal function metrics (GWAS IDs: ebi‐a‐GCST006586, ebi‐a‐GCST90025946, ieu‐a‐1097, ebi‐a‐GCST90025945, ebi‐a‐GCST003373, ebi‐a‐GCST90026654, ebi‐a‐GCST90103635) were obtained from large GWAS studies in European ancestry populations. SNPs associated with serum ketone bodies were identified via the MR Base database using a genome‐wide significance threshold (*p* < 5 × 10^−8^), and SNPs in linkage disequilibrium (within 10 000 kb or *r* ^2^ > 0.001) were excluded. Seventeen independent SNPs (SI Table S1) were selected for two‐sample MR analysis to assess the causal impact of ketone bodies on renal function.

Causal estimates were primarily calculated using the inverse variance weighted (IVW) method, which assumes no or balanced pleiotropy. Sensitivity analyses were conducted using MR‐Egger, weighted median, and weighted mode methods to provide robust estimates in the presence of potential unbalanced pleiotropy. Horizontal pleiotropy was evaluated using the MR‐Egger intercept test, and heterogeneity among SNPs was assessed via Cochran's Q statistics for both IVW and MR‐Egger estimates. When significant heterogeneity was detected, outlier‐corrected analyses were performed using MR‐PRESSO. All analyses were conducted in R version 4.4.1 using the “TwoSampleMR” R package (https://github.com/MRCIEU/TwoSampleMR).

## 3 Result

### 3.1 Cross‐Sectional Study

#### 3.1.1 Participant Characteristics

The baseline characteristics of the participants are shown in Table 1. In the final cohort of 1267 participants (median age 71 [IQR: 64–78] years; 53.5% male), 92 individuals (7.3%) had ESRD (Table 1). Notably, participants without ESRD exhibited significantly higher values for age, BMI, serum albumin, dietary intake, and DKR compared to those with ESRD (*p* < 0.05).

**TABLE 1:** Patient baseline characteristics.

Parameters	Total *n* = 1267	ESRD, *n* = 92	Non‐ESRD, *n* = 1175	*p*
Age (years)	71 (64, 78)	64 (58, 72)	72 (64, 78)	< 0.001*
Gender (male, %)	678 (53.5%)	50 (54.3%)	628 (53.4%)	0.566
Education (less than high school, %)	846 (66.8%)	58 (63.0%)	788 (67.1%)	0.355
Body mass index (kg/m^2^)	31.6 (27.7, 36.6)	30.1 (26.0, 34.5)	31.7 (27.8, 36.7)	0.009*
Smoking (%)	123 (9.7%)	4 (4.3%)	119 (10.1%)	0.082
Drinking (%)	735 (58.0%)	46 (50.0%)	689 (58.6%)	0.523
Hypertension (%)	1060 (83.7%)	80 (87.0%)	980 (83.4%)	0.890
HbA1c (%)	6.8 (6.2, 7.7)	6.5 (5.6, 7.4)	6.8 (6.2, 7.7)	0.911
Serum albumin (g/L)	40 (37, 42)	38 (34, 40)	40 (38, 42)	< 0.001*
Triglyceride (mmol/L)	1.4 (1.1, 2.1)	1.3 (0.8, 1.8)	1.4 (1.1, 2.1)	0.438
Total cholesterol (mmol/L)	4.3 (3.8, 5.2)	3.9 (3.5, 4.9)	4.3 (3.8, 5.2)	0.783
LDL cholesterol (mmol/L)	2.3 (1.8, 3.0)	2.1 (1.6, 2.5)	2.3 (1.8, 3.0)	0.693
HDL cholesterol (mmol/L)	1.2 (1.0, 1.5)	1.2 (0.9, 1.4)	1.2 (1.0, 1.5)	0.118
Dietary intake				
Protein (g)	63.4 (48.0, 81.7)	58.9 (45.2, 69.8)	63.8 (48.1, 82.6)	< 0.001*
Carbohydrate (g)	185.7 (138.9, 236.4)	164.1 (120.5, 211.7)	188.0 (141.6, 239.6)	< 0.001*
Total fat (g)	61.2 (43.8, 84.7)	51.5 (37.7, 78.3)	62.1 (44.2, 85.2)	< 0.001*
DKR value	2.7 (2.2, 3.3)	2.4 (1.9, 3.1)	2.7 (2.2, 3.3)	0.036*
DKR (%)				0.072
Q1	319 (25.2%)	19 (20.7%)	300 (25.5%)	
Q2	315 (24.9%)	29 (31.5%)	286 (24.3%)	
Q3	316 (24.9%)	24 (26.1%)	292 (24.9%)	
Q4	317 (25.0%)	20 (21.7%)	297 (25.3%)	

* Indicates that the *P* value is less than 0.05.

#### 3.1.2 DKR and ESRD

We used the Spearman correlation coefficient to analyze that DKR values were weakly negatively correlated with serum creatinine (*ρ* = −0.072, *p* = 0.011), and had no significant correlation with UACR (*ρ* = −0.022, *p* = 0.452). To refine the effect of DKR values, groups were formed according to DKR quartiles. Three logistic regression models were developed to investigate the effect of DKR on the development of DKD (Table 2), with the presence or absence of ESRD as the dependent variable. None of the three models demonstrated a statistically significant association between DKR and the risk of ESRD. In the unadjusted model 1 and the multivariate‐adjusted models 2 and 3 analyses, we observed that the risk of ESRD in the Q3 group was significantly lower than that in the Q1 group (Model 1: OR (95% CI) = 0.37 (0.16–0.85), *p* = 0.020; Model 2: OR (95% CI) = 0.32 (0.13–0.78), *p* = 0.014; Model 3: OR (95% CI) = 0.06 (0.01–0.07), *p* = 0.027). In addition, RCS analysis confirmed the inverted L‐shaped nonlinear relationship between DKR and ESRD (nonlinear = 0.018, SI Figure 1).

**TABLE 2:** ORs and 95% CI for ESRD according to quartiles of DKR.

Outcomes		Q2		Q3		Q4		DKR	
	Q1	ORs (95% CI)	*p*	ORs (95% CI)	*p*	ORs (95% CI)	*p*	ORs (95% CI)	*p*
ESRD									
Model 1	Reference	1.05 (0.51–2.16)	0.903	0.37 (0.16–0.85)	0.020*	0.57 (0.23–1.21)	0.136	0.64 (0.41–1.01)	0.058
Model 2	Reference	0.58 (0.22–1.46)	0.246	0.32 (0.13–0.78)	0.014*	0.48 (0.20–1.17)	0.101	0.69 (0.44–1.11)	0.130
Model 3	Reference	0.85 (0.29–2.48)	0.772	0.06 (0.01–0.07)	0.027*	0.27 (0.01–1.45)	0.133	0.40 (0.14–1.19)	0.109

*Note:* Model 1: unadjusted covariates. Model 2: adjusted by gender, age, education level, BMI, smoke and drink. Model 3: adjusted by gender, age, education level, BMI, smoke, drink, hypertension, TG, TC, LDL, HDL, HbA1c, serum albumin.

Abbreviations: BMI, body mass index; CI, confidence interval; DKR, dietary ketogenic ratio; ESRD, end‐stage renal disease; HDL, high‐density lipoprotein; LDL, low‐density lipoprotein; ORs, odds ratios; TC, total cholesterol; TG, triglyceride.

### 3.2 Longitudinal Observational Study

#### 3.2.1 Baseline Clinical and Pathological Features

A total of 346 patients were included in the study. At the time of renal biopsy, the mean age of the patients was 51.2 ± 9.3 years, with 70% (243/346) male, 85% (295/346) hypertensive, and 48% (165/346) having a smoking history. The mean body mass index was 25.5 ± 3.7 kg/m^2^, the mean serum albumin was 34.5 ± 7.9 g/L, and the mean hemoglobin was 118.5 ± 24.7 g/L. The median duration of diabetes mellitus in the cohort was 96 months, the median eGFR was 67.7 mL/min/1.73 m^2^, and the median urinary protein output was 5.0 g/days. Lipid profiles were as follows: triglycerides 2.3 (2.1–2.4), total cholesterol 5.3 (5.1–5.5), low‐density lipoprotein cholesterol (LDL‐C) 3.1 (2.9–3.2), and high‐density lipoprotein cholesterol (HDL‐C) 1.2 (1.0–1.5) mmol/L. Additionally, 78.5% had used RAAS inhibitors, 57.8% had used statins, and 67.9% had used insulin (Table 3).

**TABLE 3:** The baseline clinical features and pathological characteristics.

Parameters	Total *n* = 346	Quartile 1, *n* = 80	Quartile 2, *n* = 87	Quartile 3, *n* = 87	Quartile 4, *n* = 92	*p*
β‐OHB (mmol/L)	0.13 (0.08, 0.28)	0.06 (0.05, 0.07)	0.10 (0.09, 0.11)	0.20 (0.16, 0.23)	0.37 (0.31, 0.45)	
Age (years)	51.2 ± 9.3	50.5 ± 9.2	51.5 ± 9.8	51.1 ± 9.6	51.7 ± 8.6	0.710
Gender (male, %)	243 (70.2%)	57 (71.3%)	60 (69.0%)	57 (65.5%)	69 (75.0%)	0.566
Body mass index (kg/m^2^)	25.5 ± 3.7	25.4 ± 3.0	25.9 ± 4.0	25.7 ± 4.0	24.8 ± 3.5	0.551
Smoking (%)	165 (47.7%)	30 (37.5%)	40 (46.0%)	52 (59.8%)	44 (47.3%)	0.036*
Hypertension (%)	295 (85.3%)	72 (90.0%)	73 (83.9%)	72 (82.8%)	81 (85.3%)	0.572
Duration of diabetes (months)	96 (36, 132)	96 (42, 132)	120 (36, 144)	72 (36, 132)	96 (36, 141)	0.582
Fasting blood glucose (mmol/L)	8.4 ± 4.3	8.2 ± 3.3	8.1 ± 4.4	7.6 ± 3.2	9.9 ± 5.5	0.016*
HbA1c (%)	7.6 ± 1.9	7.8 ± 2.2	7.5 ± 1.7	7.8 ± 1.9	7.4 ± 1.7	0.587
e‐GFR (mL/min·1.73 m^2^)	60.3 (44.0, 91.7)	59.3 (42.5, 92.3)	57.3 (46.1, 89.2)	60.5 (43.3, 94.1)	61.6 (45.8, 92.6)	0.944
Serum creatinine (μmol/L)	115 (82, 151)	120 (76, 158)	119 (82, 146)	116 (82, 154)	113 (86, 151)	0.999
Cystatin C (μmol/L)	1.51 (1.17, 1.90)	1.54 (1.28, 1.94)	1.58 (1.16, 1.93)	1.38 (1.11, 1.75)	1.62 (1.21, 2.04)	0.047*
24 h proteinuria (g/days)	3.8 (1.8, 7.0)	3.7 (1.8, 7.7)	3.1 (1.7, 5.5)	4.3 (2.6, 8.0)	3.8 (1.4, 7.4)	0.059
Serum albumin (g/L)	34.5 ± 7.9	35.3 ± 8.3	35.1 ± 7.7	33.8 ± 6.6	33.8 ± 8.7	0.349
Hemoglobin (g/L)	118.5 ± 24.7	116.5 ± 28.3	120.7 ± 23.7	116.2 ± 23.2	120.2 ± 23.7	0.262
Triglyceride (mmol/L)	1.8 (1.3, 2.4)	1.9 (1.4, 2.7)	1.8 (1.3, 2.5)	1.8 (1.2, 2.5)	1.6 (1.3, 2.3)	0.406
Total cholesterol (mmol/L)	5.0 (4.2, 6.1)	5.0 (4.2, 6.4)	5.2 (4.2, 6.3)	5.0 (4.0, 6.1)	5.1 (4.3, 6.1)	0.851
LDL cholesterol (mmol/L)	2.9 (2.2, 3.7)	2.9 (2.2, 3.8)	2.8 (2.2, 3.7)	2.7 (2.1, 3.6)	2.9 (2.2–3.7)	0.912
HDL cholesterol (mmol/L)	1.2 (1.0, 1.5)	1.2 (0.9, 1.6)	1.3 (1.1, 1.5)	1.2 (1.0, 1.5)	1.2 (1.0, 1.6)	0.661
RAAS‐inhibitor use (%)	270 (78.5%)	59 (73.8%)	74 (86.0%)	70 (80.5%)	67 (73.6%)	0.165
Statin use (%)	200 (57.8%)	43 (53.8%)	59 (67.8%)	48 (55.2%)	50 (54.3%)	0.186
Insulin use (%)	235 (67.9%)	47 (58.8%)	59 (67.8%)	60 (69.0%)	69 (75.0%)	0.115
Renal outcome (%)	137 (39.6%)	44 (55.0%)	39 (44.8%)	28 (32.2%)	26 (28.3%)	0.001*
Pathological lesions						
RPS classification						0.249
I	15 (4.3%)	6 (7.5%)	2 (2.3%)	2 (2.3%)	5 (5.4%)	
IIa	75 (21.7%)	20 (25.0%)	18 (20.7%)	14(16.1%)	23 (25.0%)	
IIb	45 (13.0%)	8 (10.0%)	14 (16.1%)	10 (11.5%)	13 (13.7%)	
III	160 (46.2%)	33 (41.3%)	35 (40.2%)	50 (57.5%)	42 (45.7%)	
IV	51 (14.7%)	13 (16.3%)	18 (20.7%)	11 (12.6%)	9 (9.8%)	
IFTA						0.045*
0	9 (2.6%)	6 (7.5%)	2 (2.3%)	1 (1.1%)	0 (0.0%)	
1	156 (45.1%)	30 (37.5%)	37 (42.5%)	44 (50.6%)	45 (48.9%)	
2	152 (43.9%)	34 (42.5%)	44 (50.6%)	36 (41.4%)	38 (41.3%)	
3	29 (8.4%)	10 (12.5%)	4 (4.6%)	6 (6.9%)	9 (9.8%)	
Interstitial inflammation						0.090
0	15 (4.3%)	7 (8.8%)	1 (1.1%)	2 (2.3%)	5 (5.3%)	
1	254 (73.4%)	51 (63.7%)	69 (79.3%)	63 (72.4%)	71 (77.2%)	
2	77 (22.3%)	22 (27.5%)	17 (19.5%)	22 (25.3%)	16 (17.4%)	
Arteriolar hyalinosis						0.743
0	37 (10.7%)	12 (15.0%)	8 (9.2%)	7 (8.0%)	10 (10.9%)	
1	177 (51.2%)	36 (45.0%)	44 (50.6%)	47 (54.0%)	50 (54.3%)	
2	132 (38.2%)	32 (40.0%)	35 (40.2%)	33 (37.9%)	32 (34.8%)	
Arteriosclerosis						0.827
0	27 (7.8%)	4 (5.0%)	6 (6.9%)	8 (9.2%)	9 (9.8%)	
1	204 (59.0%)	45 (56.3%)	52 (59.8%)	53 (60.9%)	54 (58.7%)	
2	115 (33.2%)	31 (38.8%)	29 (33.3%)	26 (29.9%)	29 (31.5%)	

*Note:* Patients were stratified into quartiles based on baseline β‐OHB levels. Data are presented as the mean ± standard, the median or counts and percentages. Data are presented as number (%), mean ± standard deviation, or median (Q1, Q3). Correlations between the remnant cholesterol level and histopathological findings. Differences between groups were analyzed using the chi‐square test. A two‐tailed *p* < 0.05 was considered statistically significance.

Abbreviations: eGFR, estimated glomerular filtration rate; FBS, fasting blood sugar; IFTA, interstitial fibrosis and tubular atrophy; RAAS‐inhibitor, renin angiotensin aldosterone system inhibitor; RPS classification, Renal Pathology Society classification.

In terms of pathological features, we studied this cohort according to the RPS classification. For the glomerular classification, 15 (4.5%) were in class I, 75 (21.7%) in class IIa, 45 (13.0%) in class IIb, 160 (46.2%) in class III, and 51 (14.7%) in class IV. Most patients had interstitial fibrosis and tubular atrophy (IFTA) (97.4%, 337/346), interstitial inflammation (95.7%, 331/346), arteriolar hyaline degeneration (89.3%, 309/346), and arteriosclerosis (92.2%, 319/346) in their renal biopsy samples (Table 3).

#### 3.2.2 Relation Between Circulating β‐OHB Concentration and Clinicopathological Features

DKD patients were stratified into quartiles based on baseline β‐OHB levels: Q1 (0.02–0.07 mM), Q2 (0.08–0.13 mM), Q3 (0.14–0.27 mM), and Q4 (0.28–0.88 mM), comprising 23.1%, 25.1%, 25.1%, and 26.6% of the cohort, respectively (Table 3). The incidence of adverse renal outcomes declined progressively from 55.0% in Q1 to 44.8% in Q2, 32.2% in Q3, and 28.3% in Q4 (*p* = 0.001). Baseline variables including gender, age, BMI, hypertension prevalence, diabetes duration, HbA1c, eGFR, serum creatinine, albumin, hemoglobin, lipid profiles, and medication use (RAAS inhibitors, statins, insulin) did not differ significantly across groups. Notably, Q4 patients had the highest fasting glucose (9.9 ± 5.5 mmol/L, *p* = 0.016), while Q3 patients had a higher proportion of smokers (59.8%, *p* = 0.036), greater 24‐h urinary protein excretion (6.1 [4.6–7.6] g/d, *p* = 0.059), and lower serum cystatin C (1.62 [1.21–2.04], *p* = 0.047).

In addition, according to the pathologic diagnostic results in Table 3, IFTA were more severe in the Q4 group (*p* = 0.045); with no significant differences observed in glomerulopathy, arteriolar hyalinization, interstitial inflammation, or atherosclerosis among the quartiles.

#### 3.2.3 Associations Between the Circulating β‐OHB Concentration and Renal Outcomes of DKD Patients

**FIGURE 1:**
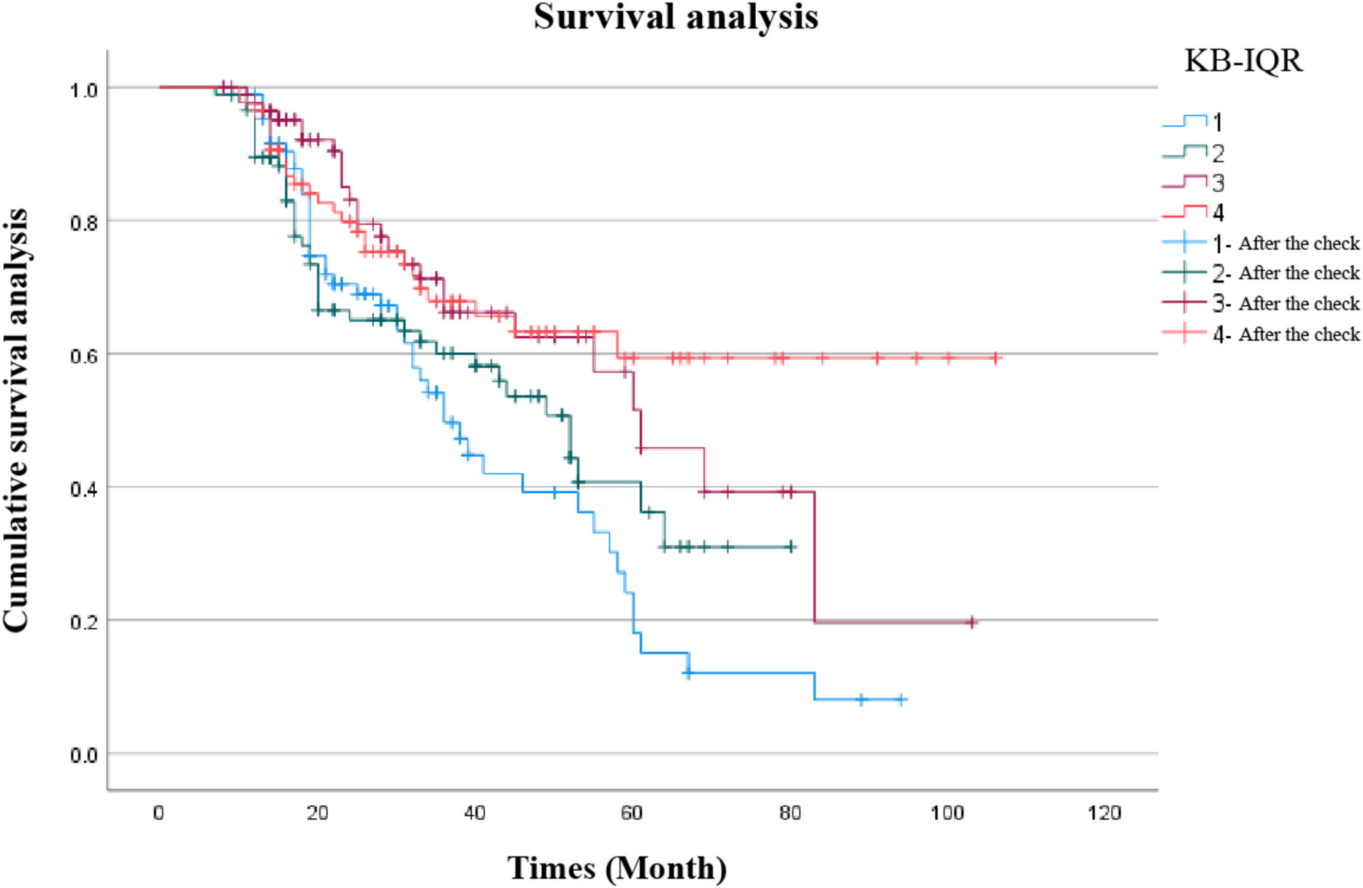
Renal survival rate between patients in four groups by Kaplan–Meier. The event‐free survival for end‐stage kidney diseases (log‐rank test, *p* = 0.004).

During a median follow‐up of 27 (17–43) months, a total of 137 (39.6%) patients achieved renal outcome. Patients in the Q4 group had a longer median renal survival time than patients in the Q1 group [31.7 (27.6–35.8) vs. 35.9 (31.1–40.7) months]. Kaplan–Meier analysis confirmed superior cumulative renal survival in Q4 (log‐rank test, *p* = 0.004; Figure 1).

Cox proportional hazards models were employed to evaluate the association between baseline β‐OHB levels and ESRD risk, using Q1 as the reference. In univariate analyses, both for continuous variables (HR = 0.18, 95% CI 0.05–0.62, *p* = 0.007) and categorical variables (HR = 0.60, 95% CI 0.38–0.97, *p* = 0.039 [Q3], HR = 0.45, 95% CI 0.28–0.74, *p* = 0.001 [Q4]), high β‐OHB level was associated with a lower risk of renal outcomes. After adjustment for age, sex, BMI, hypertension, smoking, HbA1c, fasting glucose, triglycerides, HDL, and medication use (Model 1), the Q3 (HR = 0.49, 95% CI 0.25–0.95, *p* = 0.035) and Q4 (HR = 0.50, 95% CI 0.26–0.96, *p* = 0.039) groups maintained a significantly lower ESRD risk. Further adjustment for eGFR and urinary protein excretion (Model 2) confirmed the association in Q3 (HR = 0.50, 95% CI 0.25–0.99, *p* = 0.045). Finally, after additionally correcting for pathology type (RPS classification, interstitial inflammation, and IFTA) in Model 3, the protective association in Q3 persisted (HR = 0.49, 95% CI 0.24–0.99, *p* = 0.049) (Table 4). In contrast, when modeled continuously, the association between β‐OHB and ESRD risk was not statistically significant after adjustment (Model 1: HR = 0.29, 95% CI 0.05–1.87, *p* = 0.193; Model 2: HR = 0.32, 95% CI 0.05–2.04, *p* = 0.226; Model 3: HR = 0.33, 95% CI 0.05–2.32, *p* = 0.264).

**TABLE 4:** Univariate and multivariate analysis for the association between serum β‐OHB levels and renal outcome.

Parameter		Hazard ratios (95% confidence interval) and *p*			
	Serum β‐OHB levels, median (range) (pg/mL)	Unadjusted	Model 1	Model 2	Model 3
Per 1 SD KB		0.18 (0.05–0.62)	0.29 (0.05–1.87)	0.32 (0.05–2.04)	0.33 (0.05–2.32)
		*p* = 0.007	*p* = 0.193	*p* = 0.226	*p* = 0.264
Quartile 1	0.06 (0.02–0.07)	Reference	Reference	Reference	Reference
Quartile 2	0.10 (0.08–0.12)	0.88 (0.57–1.36)	0.61 (0.34–1.11)	0.68 (0.37–1.24)	0.64 (0.35–1.20)
		*p* = 0.577	*p* = 0.108	*p* = 0.205	*p* = 0.163
Quartile 3	0.19 (0.13–0.27)	0.60 (0.38–0.97)	0.49 (0.25–0.95)	0.50 (0.25–0.99)	0.49 (0.24–0.99)
		*p* = 0.039	*p* = 0.035	*p* = 0.045	*p* = 0.049
Quartile 4	0.42 (0.28–0.88)	0.45 (0.28–0.74)	0.50 (0.26–0.96)	0.53 (0.27–1.03)	0.52 (0.26–1.03)
		*p* = 0.001	*p* = 0.039	*p* = 0.061	*p* = 0.061

*Note:* Unadjusted: univariate analysis. Model 1: adjusted for gender, age, body mass index, smoking, hypertension, HbA1c, fasting blood glucose, TG, HDL, RAAS inhibition use, insulin use and statin use. Model 2: Model 1 further adjusted for eGFR and proteinuria. Model 3: Model 2 further adjusted for pathological features.

**FIGURE 2:**
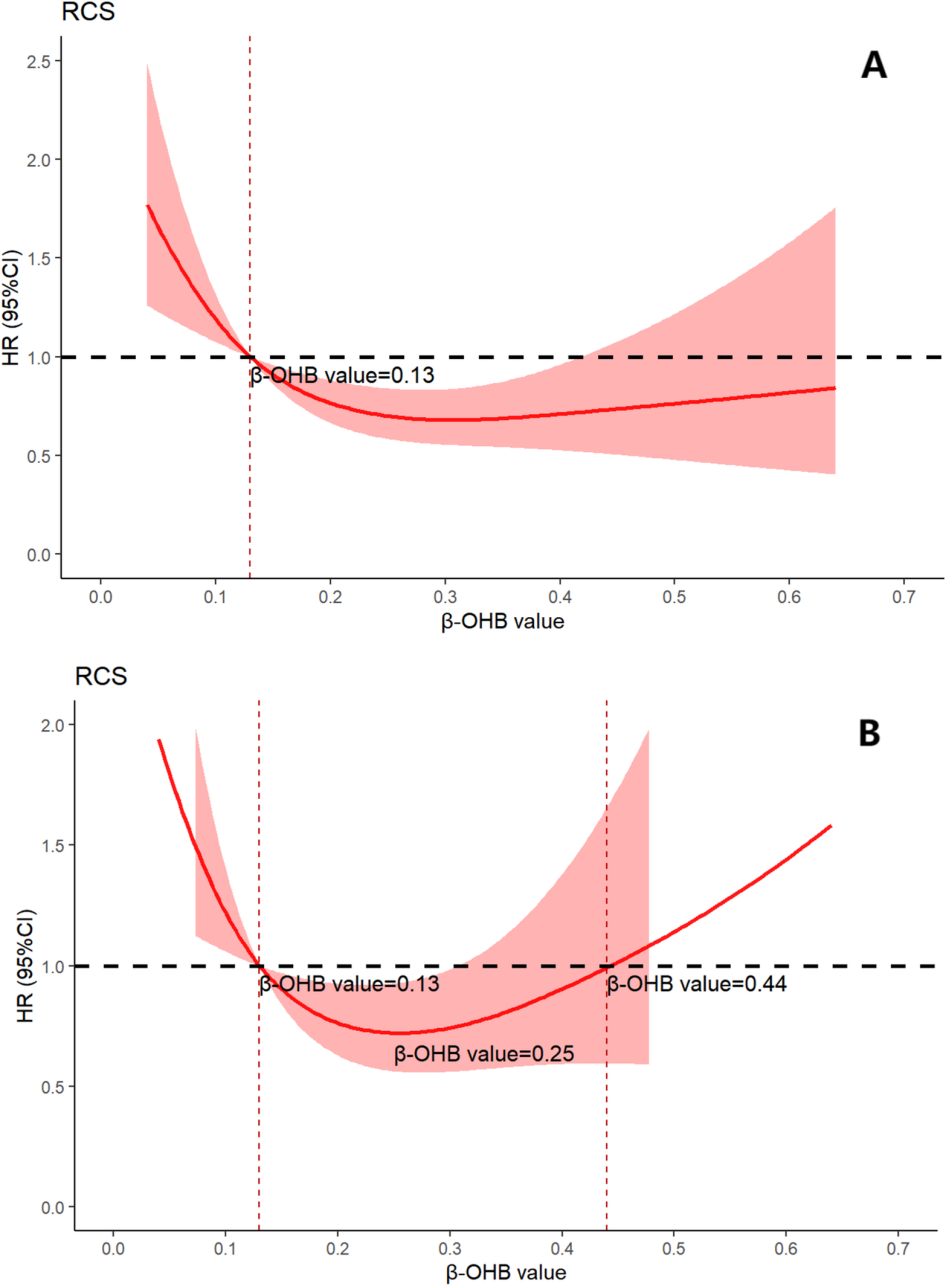
(A) Dose–response relationships of baseline serum β‐OHB with incidence of ESRD; (B) after adjusting for confounding factors. Restricted cubic spline regression model was conducted using 3 knots. The red line and red area represent the hazard radios and the 95% confidence intervals for the spline model.

RCS analysis revealed an L‐shaped relationship between serum β‐OHB and ESRD risk (nonlinear *p* = 0.01; Figure 2A), suggesting that concentrations below 0.13 mmol/L elevate ESRD risk in DKD patients. Multivariate‐adjusted RCS analysis indicated that the lowest risk of ESRD occurred at a β‐OHB concentration of 0.25 mmol/L (OR = 0.72; 95% CI, 0.57–0.92), with risk rising again at levels above 0.44 mmol/L (Figure 2B). We further divided the patients into a high β‐OHB group and a low β‐OHB group according to the cutoff value of 0.25 mmol/L. The high β‐OHB group demonstrated significantly better renal survival compared to the low β‐OHB group (log‐rank test, *p* = 0.0037; SI Figure S2). This supports the discriminative capacity of the RCS‐identified cutoff in ESRD risk.

### 3.3 MR Analysis

**FIGURE 3:**
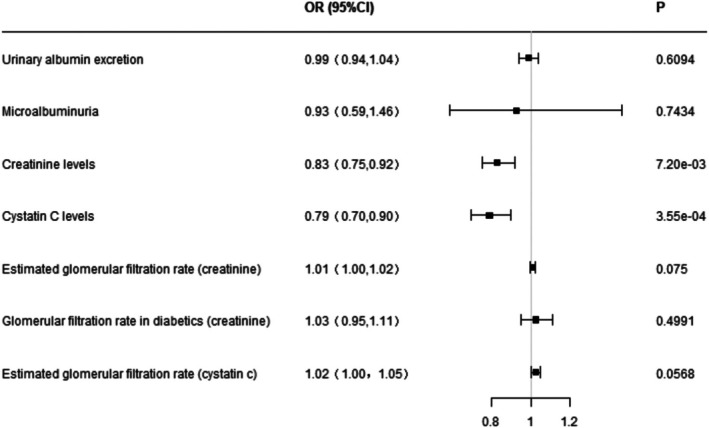
Mendelian randomization estimates for the effects of serum ketone body (3‐hydroxybutyrate) levels on renal function indicators.

In the IVW analysis, genetically predicted serum 3‐Hydroxybutyrate levels were inversely associated with both creatinine and cystatin C (*p* < 0.05). After excluding heterogeneity‐inducing SNPs via leave‐one‐out analysis and MR‐PRESSO, the results remained consistent, showing that serum 3‐Hydroxybutyrate modestly reduced creatinine by 0.995 (95% CI: 0.990–0.999; *p* = 0.025) and cystatin C by 0.985 (95% CI: 0.975–0.996; *p* = 0.009) (Figure 3). No heterogeneity was observed in the IVW and MR‐Egger analyses (SI Table S2), and no evidence of directional pleiotropy was detected (SI Table S3).

## 4 Discussion

We calculated the DKR from NHANES dietary data and explored the correlation of a KD with ESRD incidence, suggesting that a high DKR may confer renal protection by elevating serum ketone body levels. In the longitudinal analysis of biopsy‐confirmed DKD patients, higher circulating β‐OHB levels were linked to a reduced risk of ESRD, independent of conventional risk factors and medication use. Complementary MR analyses further demonstrated that genetically elevated 3‐Hydroxybutyrate levels were inversely related to markers of renal function, notably serum creatinine and cystatin C.

The kidney, a metabolically active organ relying on fatty acid oxidation and glycolysis to provide energy, is particularly vulnerable in DKD due to glucose overutilization and mitochondrial dysfunction [23, 24]. Under this condition, the β‐OHB (normally maintained at 0.02–0.27 mmol/L) serves as an important alternative energy substrate [25, 26]. The kidney preserves ketone homeostasis through synthesis, utilization, and reabsorption, with over 80% of filtered ketone bodies reclaimed by the proximal tubules [27].

Beyond energy production, KB act as signaling molecules regulating inflammation, oxidative stress, and metabolism via mTOR, AMPK (AMP‐activated protein kinase), histone deacetylases, and the NLRP3 (NOD‐like receptor family, pyrin domain containing 3) inflammasome [6, 28]. In DKD, KB help restore fatty acid oxidation and improve energy metabolism in renal tubular cells by inhibiting overactive mTORC1 [29]. β‐OHB preconditioning reduces sepsis‐induced acute kidney injury [30], inhibits vascular calcification in CKD, and alleviates oxidative stress [31].

Enhanced renal ketogenesis, as indicated by increased 3‐hydroxy‐3‐methylglutaryl‐CoA synthase 2 (HMGCS2) expression, may facilitate tissue repair in DKD [32]. KB protect both podocytes and proximal tubular cells, with emerging data implicating sodium‐coupled monocarboxylate transporter 1 (SMCT1) in maintaining ketone reabsorption; reduced SMCT1 correlates with lower serum β‐OHB, increased proteinuria, and diminished renal ATP, effects that are reversible with dietary 1,3‐butanediol [29, 33, 34]. Additionally, β‐OHB delays DKD progression by enhancing autophagy and inhibiting oxidative stress [35].

Ketogenic interventions, such as the KD, offer additional benefits by promoting weight loss, reducing inflammation, and improving insulin sensitivity [36]. In T2DM mouse models, KD has reversed diabetic kidney injury [37, 38], and our NHANES findings support that higher DKR values correlate with lower serum creatinine levels and lower ESRD incidence. Therapeutic strategies that enhance ketone body metabolism—whether through β‐OHB supplementation, KD feeding, or SGLT2i that boost endogenous ketone production—have shown promise in improving renal outcomes [38, 39].

Despite these benefits, excessive β‐OHB may be harmful. High levels can inhibit cell proliferation via the TGF‐β/Smad3 pathway, promote collagen deposition, and induce tubular hypertrophy, worsening epithelial‐mesenchymal transition [32, 40]. In hyperketonemia with hyperglycemia, oxidative and inflammatory stress may accelerate DKD progression [41, 42]. Population studies, including those based on the UK Biobank, report a J‐shaped relationship between circulating KB and DKD risk, highlighting the importance of maintaining β‐OHB within an optimal range for renal protection [12, 43, 44, 45].

Our NHANES analysis revealed that the DKR differed significantly between ESRD and non‐ESRD groups, with a lower incidence of ESRD in the Q3 DKR group—even after adjustment for confounders. Although it remains unclear whether the KD directly synchronizes changes in serum ketone body levels with DKR, potential mechanisms include enhanced metabolic health, gut microbiota modulation, and antioxidant/anti‐inflammatory effects [14, 46, 47].

In a longitudinal cohort of Chinese patients with T2DM‐DKD, we stratified participants by circulating β‐hydroxybutyrate (β‐OHB) levels. The Q1–Q3 groups patients fell into normal β‐OHB levels, and the Q4 population had KB levels outside the normal range. Patients with normal‐high β‐OHB concentrations (Q3) had the lowest incidence of ESRD, a finding that persisted after adjustment for nonmodifiable factors (age, sex, duration of diabetes), modifiable factors (HbA1c, dyslipidemia, BMI, medication history), and pathological characteristics. RCS analysis further demonstrated a nonlinear, U‐shaped relationship between β‐OHB and ESRD risk, with the lowest risk observed when β‐OHB ranged from 0.13 to 0.44 mmol/L.

In our study, patients with the highest β‐OHB levels also tended to have lower BMI and higher fasting glucose, likely reflecting the metabolic shift during lipolysis when insulin levels drop. Additionally, ketone bodies may influence IFTA, a key pathological feature of DKD and a predictor of rapid GFR decline. However, due to sample size limitations and the retrospective design, further studies with larger and more diverse populations are necessary.

MR analyses using publicly available GWAS summary statistics provided additional support for a renoprotective effect of β‐OHB. Although not all associations reached statistical significance; possibly due to the observed nonlinear relationship, the directional consistency across MR methods reinforced our longitudinal findings.

These results suggest that maintaining serum β‐OHB within a normal‐high range may independently protect against DKD progression to ESRD. Clinically, β‐OHB could serve as a biomarker for risk stratification in DKD, prompting closer monitoring in patients with levels below 0.13 mmol/L or above the normal range. Furthermore, KD interventions might offer adjunctive therapeutic benefits by optimizing KB metabolism. Given that β‐OHB has also been implicated in cardiovascular risk assessment, its incorporation into clinical management protocols for DKD may help reduce both renal and cardiovascular events [38, 41, 48].

This study has several limitations. First, our cross‐sectional analysis could not directly link DKR with serum ketone levels due to the absence of dynamic serum measurements. Second, the longitudinal study did not track temporal changes in β‐OHB, limiting insights into its dynamic relationship with DKD progression. Third, the study's small sample size and narrow range of β‐OHB values restricted the ability to evaluate the effects of elevated β‐OHB levels. Fourth, as a retrospective cohort study, some degree of selection bias was inevitable. However, the overall matching of baseline clinicopathologic characteristics across groups and the application of Cox regression analysis helped ensure that the findings remained robust. In addition, the results may only apply to patients with T2DM and biopsy‐proven DKD. Finally, our MR analysis, based on European ancestry data, calls for further GWAS studies in diverse populations to confirm these findings.

## 5 Conclusion

Ketone bodies were traditionally linked to diabetic ketoacidosis, but recent research suggests a protective role in tissue repair and kidney health. Our study found that the KD (assessed by DKR) linked with serum creatinine levels and the incidence of ESRD. We also found a nonlinear relationship between circulating β‐OHB levels and ESRD risk in ketosis‐free DKD patients, independent of other confounding factors, including age, gender, HbA1c, duration of diabetes, BMI, HDL‐C, and TG. These findings imply that maintaining circulating β‐OHB levels at normal high‐value levels may be critical for mitigating DKD progression in patients with T2DM. Further prospective cohort studies are necessary to confirm our results and guide the development of novel therapeutic strategies for DKD and other kidney diseases.

## Author Contributions

F.L. and K.L. designed the experiments. Data collection and analysis were performed by K.L., Q.Y., Y.L., Y.Z., J.Y., J.Y., J.M., and L.C. K.L. wrote the manuscript, and all authors commented on previous versions of the manuscript. All authors read and approved the final manuscript.

## Ethics Statement

The study protocol was approved by the Institutional Review Board at the West China Hospital of Sichuan University [number 2024(413)]; written informed consent was obtained from all participants.

## Consent

All patients provided informed consent. For NHANES, the protocol was approved by the National Center for Health Statistics Ethics Review Board, and written informed consent was obtained from all participants.

## Conflicts of Interest

The authors declare no conflicts of interest.

## Supporting information


**Data S1:** Supporting Information.

## Data Availability

The datasets generated during and analyzed during the current study are available from the corresponding author upon reasonable request.
