# Supplementary material for: Ketogenic Diet, Serum Ketone Bodies and Risk of End‐Stage Renal Disease in Patients With Diabetic Kidney Disease: A Multi‐Cohort Study
Source: J Diabetes. 2025 Aug 12;17(8):e70140. doi: 10.1111/1753-0407.70140 (PMC12340978; doi:10.1111/1753-0407.70140)
Supplement: Supplementary file 1 — Data S1: Supporting Information. [file JDB-17-e70140-s001.docx]

**Supplementary Information**

**Supplementary** **Table S1. ketone body(3-Hydroxybutyrate) genetic instrument summary statistics**

| SNP | chr.exposure | effect_allele.exposure | other_allele.exposure | beta.exposure | se.exposure | pval.exposure |
| --- | --- | --- | --- | --- | --- | --- |
| rs10127775 | 1 | T | A | -0.0302326 | 0.00422503 | 8.30E-13 |
| rs585188 | 5 | A | G | -0.0459785 | 0.00473538 | 2.70E-22 |
| rs9987289 | 8 | G | A | 0.0654579 | 0.0071752 | 7.30E-20 |
| rs2645433 | 8 | C | G | -0.0276302 | 0.00426017 | 8.80E-11 |
| rs4149307 | 9 | T | C | 0.0347025 | 0.00566933 | 9.30E-10 |
| rs2575876 | 9 | A | G | -0.0316957 | 0.00475019 | 2.50E-11 |
| rs2419604 | 10 | G | A | 0.0409399 | 0.00461202 | 6.90E-19 |
| rs964184 | 11 | C | G | -0.0486628 | 0.00608193 | 1.20E-15 |
| rs1169297 | 12 | A | G | -0.0242485 | 0.00436352 | 2.70E-08 |
| rs12314700 | 12 | T | A | -0.0350217 | 0.00532395 | 4.80E-11 |
| rs72702354 | 14 | G | A | -0.068998 | 0.0103685 | 2.80E-11 |
| rs28929474 | 14 | T | C | -0.158295 | 0.0147558 | 7.50E-27 |
| rs473224 | 15 | G | T | 0.0340063 | 0.00591221 | 8.80E-09 |
| rs35853021 | 15 | T | G | -0.0292474 | 0.00430506 | 1.10E-11 |
| rs9930957 | 16 | T | C | 0.0632021 | 0.00562402 | 2.70E-29 |
| rs117643180 | 17 | A | C | -0.0849923 | 0.0130808 | 8.20E-11 |
| rs429358 | 19 | C | T | 0.054874 | 0.00571035 | 7.30E-22 |

**Supplementary Table S2a. MR heterogeneity test (MR-Egger)**

| Exposure | Outcome | N of SNPs | Q statistic | Q df | Q p-value |
| --- | --- | --- | --- | --- | --- |
| 3-Hydroxybutyrate | Urinary albumin excretion | 11,684,850 | 18.779 | 11 | 0.065 |
|  | Microalbuminuria | 2,191,461 | 5.882 | 6 | 0.436 |
|  | Creatinine levels | 4,232,009 | 6.602 | 5 | 0.356 |
|  | Cystatin C levels | 4,232,088 | 5.911 | 4 | 0.206 |
|  | Estimated glomerular filtration rate (creatinine) | 8422080 | 5.803 | 7 | 0.563 |
|  | Glomerular filtration rate in diabetics (creatinine) | 2169300 | 6.426 | 6 | 0.377 |
|  | Estimated glomerular filtration rate (cystatin c) | 7250211 | 10.962 | 9 | 0.278 |

**Supplementary Table S2b. MR heterogeneity test (IVW)**

| Exposure | Outcome | N of SNPs | Q statistic | Q df | Q p-value |
| --- | --- | --- | --- | --- | --- |
| 3-Hydroxybutyrate | Urinary albumin excretion | 11,684,850 | 17.547 | 12 | 0.071 |
|  | Microalbuminuria | 2191461 | 6.952 | 7 | 0.434 |
|  | Creatinine levels | 4,232,009 | 6.791 | 6 | 0.476 |
|  | Cystatin C levels | 4232088 | 6.670 | 5 | 0.246 |
|  | Estimated glomerular filtration rate (creatinine) | 8422080 | 9.242 | 8 | 0.322 |
|  | Glomerular filtration rate in diabetics (creatinine) | 2169300 | 6.428 | 7 | 0.491 |
|  | Estimated glomerular filtration rate (cystatin c) | 7250211 | 13.378 | 10 | 0.203 |

**Supplementary Table S3. MR directional pleiotropy test**

| Exposure | Outcome | Egger intercept | SE | p value |
| --- | --- | --- | --- | --- |
| 3-Hydroxybutyrate | Urinary albumin excretion | 0.0026 | 0.003 | 0.401 |
|  | Microalbuminuria | -0.042 | 0.041 | 0.341 |
|  | Creatinine levels | 0.0043 | 0.013 | 0.752 |
|  | Cystatin C levels | 0.014 | 0.0062 | 0.057 |
|  | Estimated glomerular filtration rate (creatinine) | -0.001 | 0.001 | 0.236 |
|  | Glomerular filtration rate in diabetics (creatinine) | 0.001 | 0.007 | 0.962 |
|  | Estimated glomerular filtration rate (cystatin c) | -0.001 | 0.001 | 0.464 |


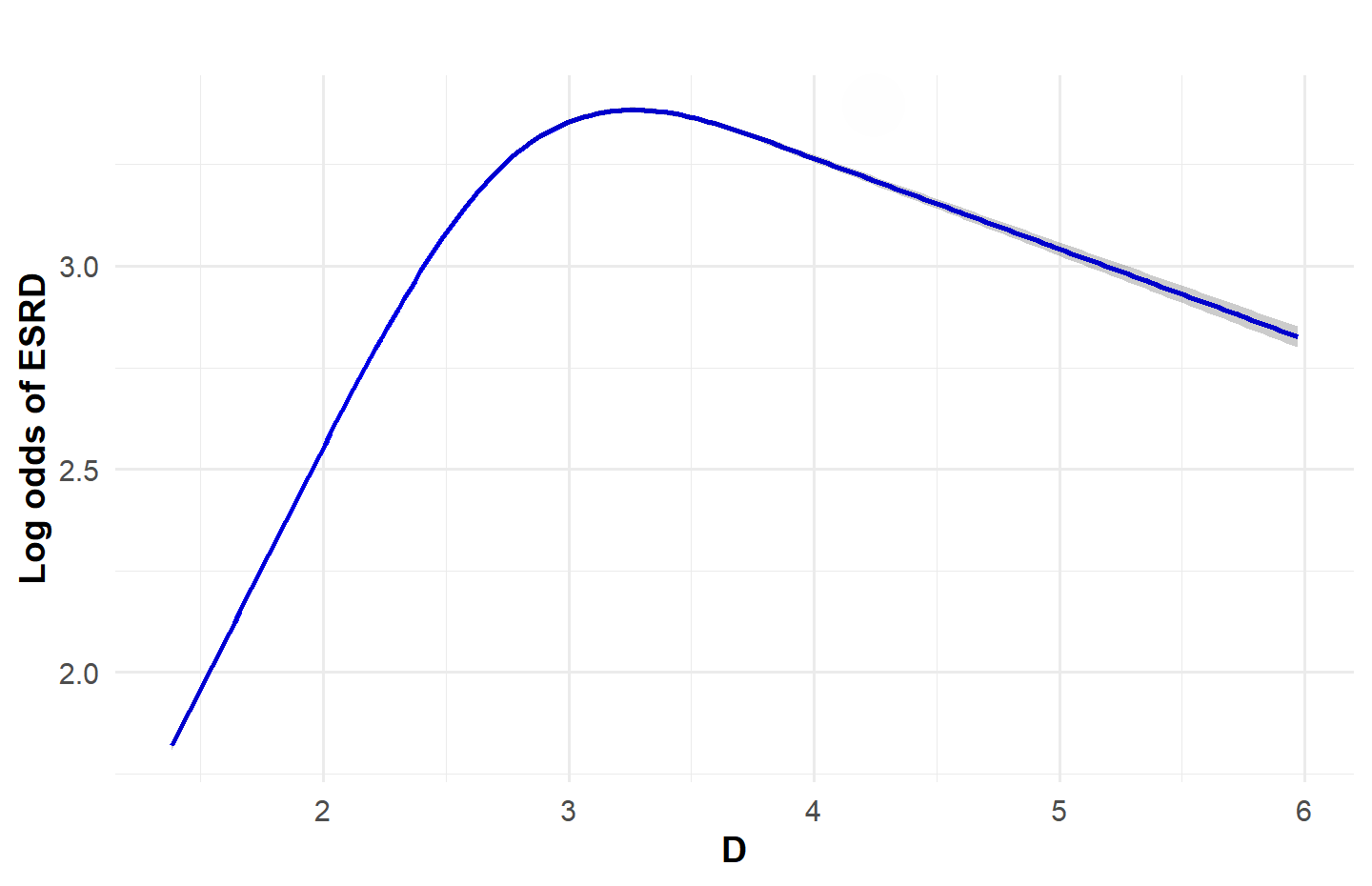


**Supplementary Figure S1.** Non-linear relationships between DKR and ESRD. Restricted cubic spline regression model was conducted using 3 knots.


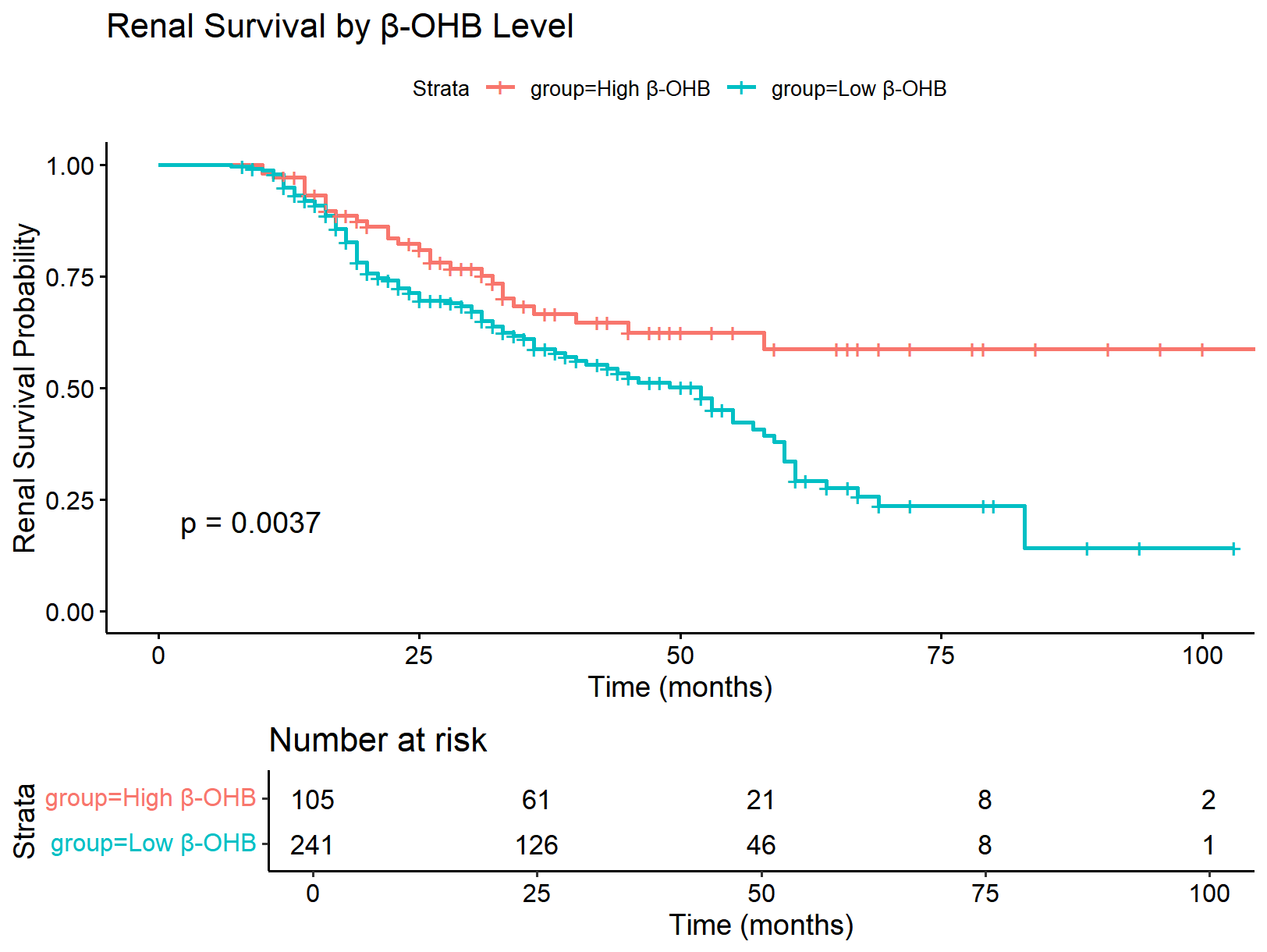


**Supplementary Figure S2.** Kaplan-Meier renal survival curves stratified by baseline β-OHB levels.
